# Wheat Phosphates

**Published:** 1879-06

**Authors:** Elwood C. Lester

**Affiliations:** Penna


### WHEAT PHOSPHATES.

BY ELWOOD C. LESTER, M. D., PENNA.

The value of wheat as an aliment has long been recognized, and the fact that a very large per cent. of the nutrient properties of this cereal resides in its cortical portion is fully conceded, but it has been less than a dozen years, since England's distinguished physician, Dr. Tilbury Fox, isolated the phosphates from the wheat bran and presented them to the profession in an assimilable form. While there is no doubt that our food, properly selected and prepared, contains all the phosphorous elements the human organism requires, yet owing to the enfeebled condition of the digestive process, these phosphates are not assimilated from the food, and the organism suffers the consequences.

The hypophosphites manufactured in the laboratory, although of value in phthisis and nervous prostration, are not equal, in the maladies of pregnancy, lactation and childhood, to the phosphates elaborated in the germinal portions of wheat.

"There is something special," as Dr. Tilbury Fox remarks "in the phosphates that have been produced in a vegetable organism in contrast to those found in the laboratory. It is not the amount, but the *kind*, which determines the result, and evinces the physiological action of the phosphates in pathological conditions accruing from their deficiency." This view of the introducer of wheat phosphates into medical practice is sustained and corrobated by Drs. Andre, Sanson, Routh, Hammond and Polk, and I believe will not find to-day any intelligent opponent.

I have frequently administered wheat phosphates to children who have been fretful, restless and unable to sleep and have found a quiet, amiable disposition, a good appetite, excellent digestion and natural sleep follow their use.

The wheat phosphates, now made after Dr. Fox's formula, the organic phosphates and a combination of the phophorus elements of the inner portion of the wheat shell, mixed with three portions of malt sugar, and the wine of wheat are all desirable forms in which to administer these remedies.

I give children, who teeth hard, whose nutrition is faulty, who are scrofulous, who have rickets, are sleepless, restless, irritable and become thin, the "Wine of Wheat" in doses from half a teaspoonful to a teaspoonful thrice daily and the result has been all that could be desired. During pregnancy and lactation, women who have trifacial neuraligia, backache, toothache, decay of the teeth and general deterioration of health will seldom fail of finding prompt and permanent relief, if some of the above forms of the phosphates are used.

